# Association between phosphate disturbances and mortality among critically ill patients with sepsis or septic shock

**DOI:** 10.1186/s40360-021-00487-w

**Published:** 2021-05-28

**Authors:** Shmeylan A. Al Harbi, Hasan M. Al-Dorzi, Albatool M. Al Meshari, Hani Tamim, Sheryl Ann I. Abdukahil, Musharaf Sadat, Yaseen Arabi

**Affiliations:** 1 Pharmaceutical Care Department, Ministry of National Guard Health Affairs, Riyadh, Saudi Arabia; 2 King Abdullah International Medical Research Center, Riyadh, Saudi Arabia; 3 College of Pharmacy, King Saud bin Abdulaziz University for Health Sciences, Riyadh, Saudi Arabia; 4 Intensive Care Department, Ministry of National Guard Health Affairs, Riyadh, Saudi Arabia; 5 College of Medicine, King Saud bin Abdulaziz University for Health Sciences, Riyadh, Saudi Arabia; 6 Saudi Food and Drug Authority, Riyadh, Saudi Arabia; 7 Biostatistics and Bioinformatics, King Abdullah International Medical Research Center, Riyadh, Saudi Arabia; 8 King Saud bin Abdulaziz University for Health Sciences, Riyadh, Saudi Arabia; 9 Department of Internal Medicine, American University of Beirut- Medical Center, Beirut, Lebanon

**Keywords:** Phosphate, Mortality, Sepsis, Septic shock, Critically ill, Intensive care, Saudi Arabia

## Abstract

**Objective:**

The aim of this study is to examine the association of hypophosphatemia and hyperphosphatemia on the first day of ICU admission with mortality in septic critically ill patients.

**Methods:**

In this retrospective cohort study, all adult patients who were admitted to the medical-surgical ICUs between 2014 and 2017 with sepsis or septic shock were categorized as having hypophosphatemia, normophosphatemia and hyperphosphatemia based on day 1 serum phosphate values. We compared the clinical characteristics and outcomes between the three groups. We used multivariate analysis to examine the association of hypophosphatemia and hyperphosphatemia with these outcomes.

**Results:**

Of the 1422 patients enrolled in the study, 188 (13%) had hypophosphatemia, 865 (61%) normophosphatemia and 369 (26%) had hyperphosphatemia. The patients in the hyperphosphatemia group had significantly lower GCS, higher APACHE II scores, higher serum creatinine, increased use of vasopressors, and required more mechanical ventilation with lower PaO2/FiO2 ratio compared with the other two groups. In addition, the hyperphosphatemia group showed significantly higher ICU and hospital mortality in comparison with the other two groups.

**Conclusion:**

Hyperphosphatemia and not hypophosphatemia on the first ICU admission day was associated with an increase in the ICU and hospital mortality in septic critically ill patients.

## Background

Acute critical illness predisposes patients to serum phosphate disturbances [1–6], whether hypo or hyperphosphatemia. In these patients, hypophosphatemia may develop as a result of decreased intake or absorption, increased renal excretion, and/ or internal redistribution. It can be seen in patients with hyperventilation, respiratory alkalosis, insulin secretion and provision, hungry bone syndrome and refeeding syndrome [7]. On the other hand, hyperphosphatemia occurs as a consequence of renal dysfunction, iatrogenic administration of large phosphate load, tumor lysis syndrome, hemolysis, rhabdomyolysis or lactic ketoacidosis [8].

The reported prevalence of hypophosphatemia in critically ill patients varies widely across different studies and ranges between 10 and 80% [9–11]. It is particularly high in sepsis. Hypophosphatemia is considered one of the early findings of sepsis. Studies have shown that hypophosphatemia occurs in up to 80% of septic patients and is associated with very high levels of tumor necrosis factor-alpha and interleukin (IL)-6 and of soluble IL receptor-2R and IL-6R, especially in those patients with positive blood cultures [10, 12]. Hyperphosphatemia is also common in the intensive care unit [13]. In a study of 2700 of ICU patients, 45% of more than 10,000 serum phosphate measurements were indicative of hyperphosphatemia [7]. Another study found that hyperphosphatemia was independently associated with 28-day in-hospital mortality [14]. Sepsis, which is commonly associated with acute kidney injury, is also a risk factor for hyperphosphatemia.

Serum phosphate disturbances have been associated with adverse clinical outcomes, such as the need for prolonged mechanical ventilation [15–18], cardiac dysfunction and arrhythmias [9, 19], hematologic dysfunction, and insulin resistance [1]. The evidence on the clinical significance of serum phosphate disturbances in patients with sepsis remains scarce and controversial. It is unclear if phosphate disturbances are independently associated with mortality in septic patients or they represent markers for higher severity of illness. Thus, we sought to examine the association of hypophosphatemia and hyperphosphatemia with mortality among septic patients.

## Methods

### Setting

This is a retrospective cohort study that was conducted in the adult medical-surgical ICUs of King Abdulaziz Medical City, which is a tertiary-care academic referral hospital in Riyadh, Saudi Arabia. The ICU admits medical and surgical patients and operates as a closed unit with onsite coverage by critical care board-certified intensivists 24 h per day, 7 days per week [20]. The nurse-to-patient ratio in the unit is approximately 1:1.2 [20]. In addition, clinical pharmacists are a part of the daily multidisciplinary rounds. The ICU has an electrolyte replacement protocol for hypokalemia, hypophosphatemia and hypomagnesemia. The dose of replaced electrolyte depends on the respective serum level taking into consideration kidney function and weight.

This study was approved by the Institutional Review Board of the Ministry of National Guard Health Affairs, Riyadh, Saudi Arabia.

### Participants

All adult patients who were admitted to the medical-surgical ICUs between January 2014 and September 2017 were screened for the following inclusion criteria: age ≥ 18 years old, sepsis or septic shock on ICU admission, and expected ICU length of stay (LOS) > 24 h. Owing to the retrospective nature of the study, no sample size calculation was done.  Sepsis was defined using the sepsis-3 definition as the presence of an infection with signs of organ dysfunction, which were represented by a Sequential Organ Failure Assessment (SOFA) score of 2 points or greater. On the other hand, septic shock was defined as the subset of sepsis with a vasopressor requirement to maintain the mean arterial pressure [21] of 65 mmHg or greater and a serum lactate level greater than 2 mmol/L (> 18 mg/dL) in the absence of hypovolemia [22]. In our ICU, the initial fluid of choice for resuscitation was crystalloid such as normal saline or lactated ringer in a dose of approximately 30 ml/kg [23]. Exclusion criteria included admission to the Burn Unit, pregnancy or receiving parenteral nutrition, vitamin D preparations, or phosphate binders. Cardiac patients, including those admitted with ST-elevation myocardial infraction were admitted to cardiac ICUs and hence were not included in this study.

### Data collection

The following data were extracted from the prospectively collected ICU database: age, gender, admission category (medical, surgical, and non-operative trauma or non-operative and post-operative), Acute Physiology and Chronic Health Evaluation (APACHE II) score [24], Glasgow Coma Scale (GCS) [25], chronic comorbidities (chronic liver disease, chronic cardiovascular disease, chronic respiratory disease, chronic renal disease and chronic immunosuppression) as defined by the APACHE system, history of diabetes mellitus, presence of sepsis or septic shock on admission, presence of acute kidney injury [22, 26], need for mechanical ventilation and vasopressor use. We also documented the admission serum creatinine level, International Normalized Ratio (INR) and platelet count. Furthermore, all phosphate level data were collected from the hospital information system BESTCare (BESTCare 2.0, Seoul, South Korea). Phosphate levels are routinely measured in the chemistry lab using phosphomolybdate method by Abbott Alinity ci series (Abbott Park, Illinois, U.S.A). Blood sample is delivered to the chemistry lab via pneumatic system within 5–10 min of sample collection by the ICU primary nurse. 

Normophosphatemia is commonly defined as total serum phosphate of 0.80 to 1.45 mmol/L (2.5 to 4.5 mg/dl) [1, 27]. The patients in this study were divided into three groups based on their serum phosphate level during the first 24 h of ICU admission. The normophosphatemia group was defined as a patient with phosphate level of 0.74 to 1.52 mmol/L, while hypophosphatemia less than 0.73 mmol/L, and hyperphosphatemia more than 1.52 mmol/L. These cutoffs were selected based on the thresholds for phosphate replacement in the ICU electrolyte replacement protocol and the hospital laboratory reference values.

### Outcomes

The primary outcomes were ICU and hospital mortality. The secondary outcomes were mechanical ventilation duration and ICU and hospital length of stay.

### Statistical analysis

Statistical analysis was performed using the Statistical Analysis Software (SAS, Release 8, SAS Institute Inc., Cary, NC, 1999, USA). Baseline characteristics, interventions and outcomes were reported as numbers with percentages for categorical variables and as medians with the first and third quartiles (Q1 and Q3, respectively) for continuous variables. They were compared among groups using the Chi-square test and ANOVA, respectively.

To determine if phosphate level was an independent predictor for hospital mortality, multivariable logistic regression analysis was performed with the normophosphatemia group as the reference. The variables included in the model were those known to be clinically relevant (age, APACHE II, sex, serum creatinine). Results were presented as adjusted odds ratio (aOR) with 95% confidence interval (CI).

We carried out subgroup analyses with stratification by the following variables: age, sepsis, diabetes, vasopressor use, operative admission category, chronic cardiac, respiratory and liver disease, chronic immunosuppression, acute kidney injury, and hypertension, adjusting for the same clinically relevant covariates mentioned above. Tests of interaction were performed to assess whether these variables were effect modifiers of the association between phosphate level and mortality. A *p*-value ≤0.05 was considered statistically significant.

## Results

### Patient characteristics

Of the 1422 patients enrolled in the study, 188 (13%) were categorized as hypophosphatemia with a median of 0.6 mmol/L, 865 (61%) as normophosphatemia with a median of 1.09 mmol/L and 369 (26%) as hyperphosphatemia with a median of 1.9 mmol/L, at day one of their ICU admission. Table 1 presents the baseline characteristics of the hypophosphatemia, normophosphatemia and hyperphosphatemia groups.

**Table 1:** Baseline and clinical characteristics of patients with hypophosphatemia (Phosphate < 0.74 mmol/L)), normophosphatemia (Phosphate 0.74-1.52 mmol/L) and hyperphosphatemia (Phosphate > 1.52 mmol/L)

Variable	Hypophosphatemia group ***N*** = 188	Normophosphatemia group ***N*** = 865	Hyperphosphatemia group ***N*** = 369	***p*** value
**Age (years), median (Q1, Q3)**	62 (42, 75)	67 (50, 76)	66 (56, 75)	0.09
**Female sex, n (%)**	87 (46.3)	353 (40.8)	156 (42.3)	0.38
**Admission category, n (%)**				
**Medical**	185 (98.4)	849 (98.2)	362 (98.1)	0.94
**Surgical**	3 (1.6)	15 (1.7)	6 (1.6)	
**Non-operative trauma**	0 (0)	1 (0.12)	1 (0.3)	
**APACHE II, median (Q1, Q3)**	19 (15, 24)	21 (16, 26)	26 (21, 30)	< 0.0001
**Mechanical ventilation, n (%)**	83 (44.2)	411 (47.5)	233 (63.1)	< 0.0001
**GCS, median (Q1, Q3)**	14 (10, 15)	14 (10, 15)	13 (8, 15)	0.006
**Vasopressor, n (%)**	84 (44.7)	406 (46.9)	242 (65.6)	< 0.0001
**PaO**_**2**_**/FiO**_**2**_ **ratio, median (Q1, Q3)**	216 (132, 316)	216 (132, 316)	171 (108, 361)	< 0.0001
**Chronic comorbidities- n (%)**				
**Chronic respiratory disease, n (%)**	7 (3.7)	56 (6.5)	17 (4.6)	0.20
**Chronic cardiac disease, n (%)**	30 (15.9)	252 (29.1)	133 (36.0)	< 0.0001
**Chronic liver disease, n (%)**	14 (7.5)	48 (5.6)	36 (9.8)	0.03
**Chronic immunosuppression, n (%)**	43 (22.9)	177 (20.5)	58 (15.7)	0.07
**Chronic renal disease, n (%)**	4 (2.1)	19 (2.2)	25 (6.8)	0.0001
**Acute Kidney Injury, n (%)**	14 (7.5)	144 (16.7)	132 (35.8)	< 0.0001
**Renal replacement therapy**	8 (4.3)	64 (7.4)	78 (21.1)	< 0.0001
**Creatinine (**μmol**/L), median (Q1, Q3)**	67 (50,98)	90 (60,154)	188 (111, 302)	< 0.0001
**INR, median (Q1, Q3)**	1.3 (1.1, 1.6)	1.3 (1.1, 1.6)	1.5 (1.2, 2)	< 0.0001
**Platelet (10**^**9**^**/L), median (Q1, Q3)**	164 (89, 255)	203 (119.5, 308)	164 (90, 289)	< 0.0001
**Average phosphate level (mmol/L), median (Q1, Q3)**	0.6 (0.5, 0.7)	1.09 (0.9, 1.3)	1.9 (1.7, 2.2)	< 0.0001
**Lactic acid (μmol/L), median (Q1,Q3)**	2.2 (1.3, 4.3)	1.9 (1.2, 3.3)	3.09 (1.5, 6.9)	< 0.0001
**Estimated GFR, median (Q1, Q3)**	93.6 (58.5, 139.5)	70.4 (37.2, 117.1)	27.6 (17.6, 54.1)	< 0.0001

*APACHE* Acute Physiology and Chronic Health Evaluation, *GCS* Glasgow Coma Scale, *INR* International Normalized Ratio, *Q1* the first quartile, *Q3* the third quartile

Among the three groups, patients who had hyperphosphatemia had significantly lower GCS (13 (8, 15)), higher APACHE II scores (26 (21, 30)), higher serum creatinine (188 (111,302) μmol/L), higher use of vasopressors (65.6%), and required more mechanical ventilation (63.1%) with lower PaO2/FiO2 ratio 171 (108, 361; *p* < 0.0001). Also, chronic cardiac and renal disease were significantly more prevalent in the hyperphosphatemia group (*p* < 0.0001). Moreover, the hyperphosphatemia group had significant coagulopathy with lower platelet count than the other two groups (*p* < 0.0001).

### Outcomes

Table 2 presents the outcomes of the three patient groups. The hyperphosphatemia group showed significantly higher ICU mortality (114 (32.0%)) and hospital mortality (165 (44.7%)) when compared with hypophosphatemia and normophosphatemia group (*p* < 0.0001). The median hospital length of stay was higher in the hypophosphatemia group (22 days; Q1, Q3: 7, 51) and normophosphatemia group (22 days; Q1, Q3: 12, 51) than those with hyperphosphatemia (17 days; Q1, Q3: 7, 39). In contrast, mechanical ventilation duration was longer in the hyperphosphatemia group (2 days; Q1, Q3: 0, 6 versus 0; Q1, Q3: 0, 4 for hypophosphatemia patients). However, among survivors only, there was no difference in the ICU length of stay, hospital length of stay and ventilation duration between the three groups.

**Table 2:** Outcomes of patients with hypophosphatemia, normophosphatemia and hyperphosphatemia

Variable	Hypophosphatemia group ***N*** = 188	Normophosphatemia group ***N*** = 865	Hyperphosphatemia group ***N*** = 369	***p***-value
**Categorical outcomes**				
**ICU mortality, n (%)**	21 (11.4)	143 (17.1)	114 (32.0)	< 0.0001
**Hospital mortality, n (%)**	44 (23.5)	226 (26.2)	165 (44.7)	< 0.0001
**Continuous outcomes in all patients**				
**ICU length of stay (days), median (Q1, Q3)**	3.1 (0.83, 9.3)	3.9 (1.04, 10.04)	2.8 (0.7, 10)	0.09
**Hospital length of stay (days), median (Q1, Q3)**	22 (7, 51)	22 (12, 51)	17 (7, 39)	0.001
**Mechanical ventilation duration (days), median (Q1, Q3)**	0 (0, 4)	1 (0, 6)	2 (0, 6)	0.005
**Continuous outcomes in survivors**				
**ICU length of stay (days), median (Q1, Q3)**	2.8 (0.7, 8.5)	3.3 (0.9, 9.1)	2.7 (0.4, 9.9)	0.42
**Hospital length of stay (days), median (Q1, Q3)**	22 (10, 53)	22 (12, 52)	26.5 (14.5, 65)	0.46
**Mechanical ventilation duration (days), median (Q1, Q3)**	0 (0, 4)	0 (0, 3)	1 (0, 4)	0.21

*ICU* intensive care unit, *Q1* the first quartile, *Q3* the third quartile

On multivariate logistic regression analysis, hyperphosphatemia was found to be significantly associated with ICU mortality (aOR 1.6, 95% CI: 1.13–2.28, *p* = 0.008) and hospital mortality (aOR 1.7, 95% CI: 1.21–2.29, *p* = 0.002, respectively) when compared to normophosphatemia group. However, there was no association between ICU and hospital mortality with hypophosphatemia and normophosphatemia patients (ICU mortality: aOR 0.60, 95% CI: 0.33–1.1, *p* = 0. 08; hospital mortality: aOR 0.89, 95% CI: 0.57–1.38, *p* = 0.59) (Table 3).

**Table 3:** Multivariate logistic regression analysis of the association of phosphate levels and mortality

	Hypophosphatemia group vs Normophosphatemia group			Hyperphosphatemia group vs Normophosphatemia group		
	aOR	95% CI	***p***-value	aOR	95% CI	***p***-value
**ICU mortality**	0.60	0.33–1.1	0.08	1.6	1.13–2.28	0.008
**Hospital mortality**	0.89	0.57–1.38	0.59	1.7	1.21–2.29	0.002

*aOR* adjusted odds ratio, *CI* confidence interval

Tables 4 and 5 show the association between phosphate levels and all-cause ICU or hospital mortality in several subgroups of patients. The multivariant analysis showed that among non-liver disease hyperphosphatemia patients were associated with higher ICU mortality (aOR 1.68, 95% CI: 1.61–2.42), *p* = 0.007) compared with normophosphatemia group.

**Table 4:** Multivariate analysis of the association of phosphate levels and ICU mortality in selected subgroups of patients

	Hypophosphatemia group		Hyperphosphatemia group		
Variable	aOR (95% CI)	***P***-value	aOR (95% CI)	***P***-value	***P***-value for interaction
**Age**					
Age < 67 years	0.74 (0.36–1.50)	0.40	1.32 (0.80–2.20)	0.29	0.69
Age > 67 years	0.35 (0.12–1.04)	0.06	1.84 (1.11–1.04)	0.02	
**Sex**					
Male	0.56 (0.27–1.18)	0.13	1.79 (1.15–2.81)	0.01	0.65
Female	0.65 (0.25–1.68)	0.38	1.35 (0.76–2.39)	0.31	
**Chronic respiratory disease**					
Yes	< 0.001 (< 0.001- > 999.9)	0.96	1.69 (0.37–7.8)	0.50	0.27
No	0.63 (0.35–1.13)	0.12	1.58 (1.10–1.14)	0.01	
**Chronic cardiac disease**					
Yes	0.54 (0.15–1.96)	0.35	2.23 (1.23–4.05)	0.008	0.48
No	0.58 (0.30–1.12)	0.11	1.36 (0.87–2.11)	0.18	
**Chronic renal disease**					
Yes	< 0.001 (< 0.001- > 999.9)	0.96	1.82 (0.42–7.94)	0.43	0.97
No	0.63 (0.35–1.14)	0.13	1.16 (1.11–2.30)	0.01	
**Chronic liver disease**					
Yes	0.39 (0.05–3.15)	0.37	0.55 (0.13–2.25)	0.41	0.007
No	0.62 (0.33–1.14)	0.12	1.68 (1.16–2.42)	0.006	
**Chronic immunosuppression**					
Yes	0.72 (0.27–1.94)	0.52	1.93 (0.86–4.40)	0.52	0.19
No	0.51 (0.24–1.07)	0.08	1.72 (1.15–2.56)	0.008	
**APACHE II score**					
< 23	0.62 (0.27–1.43)	0.26	1.97 (1.07–3.62)	0.03	0.78
> 23	0.57 (0.25–1.30)	0.18	1.51 (0.98–2.33)	0.06	
**Diabetes**					
Yes	0.51 (0.17–1.53)	0.23	1.66 (0.96–2.88)	0.07	0.90
No	0.60 (0.30–1.20)	0.15	1.36 (0.83–2.22)	0.22	
**Vasopressors**					
Yes	0.58 (0.27–1.25)	0.16	1.45 (0.95–2.21)	0.09	0.89
No	0.65 (0.26–1.62)	0.36	1.71 (0.89–3.29)	0.11	
**ICU duration (days)**					
≤ 5	0.71 (0.26–1.95)	0.50	1.35 (0.70–2.60)	0.37	0.59
> 5	0.58 (0.27–1.23)	0.16	1.46 (0.92–2.30)	0.11	
**Estimated GFR**					
≤ 73 ml/min	0.61 (0.24–1.57)	0.30	1.72 (1.15–2.58)	0.009	0.18
> 73 ml/min	0.60 (0.28–1.28)	0.18	1.04 (0.45–2.41)	0.93	
**Acute Kidney Injury**					
Yes	0.39 (0.07–2.14)	0.28	1.49 (0.79–2.82)	0.22	0.53
No	0.63 (0.34–1.17)	0.14	1.56 (1.02–2.40)	0.04	
**Type of admission**					
Non-operative	0.55 (0.30–1.00)	0.05	1.6 (1.10–1.14)	0.01	0.89
Post-operative	14 (0.45–434)	0.13	4.70 (0.22–97.50)	0.32	

*aOR* adjusted odds ratio, *APACHE* Acute Physiology and Chronic Health Evaluation, *CI* confidence interval, *GFR* glomerular filtration rate, *ICU* intensive care unit

**Table 5:** Multivariate analysis of the association of phosphate levels and hospital mortality in selected subgroups of patients

	Hypophosphatemia group		Hyperphosphatemia group		
Variable	aOR (95% CI)	***p*** value	aOR (95% CI)	***p***-value	***p***-value for interaction
**Age (years)**					
Age < 67	0.93 (0.52–1.68)	0.81	1.60 (1.01–2.53)	0.05	0.17
Age > 67	0.84 (0.43–1.66)	0.62	1.71 (1.10–2.70)	0.02	
**Sex**					
Male	0.95 (0.54–1.64)	0.84	1.54 (1.02–2.32)	0.04	0.17
Female	0.80 (0.38–1.68)	0.56	1.87 (1.11–3.13)	0.02	
**Chronic respiratory disease**					
Yes	0.48 (0.05–4.53)	0.52	1.62 (0.40–6.49)	0.50	0.29
No	0.93 (0.59–1.46)	0.74	1.64 (1.17–2.30)	0.004	
**Chronic cardiac disease**					
Yes	1.33 (0.53–3.34)	0.54	1.35 (0.74–2.46)	0.33	0.45
No	0.76 (0.46–1.27)	0.30	1.68 (1.14–2.50)	0.01	
**Chronic renal disease**					
Yes	0.88 (0.06–13.28)	0.92	1.25 (0.30–5.41)	0.76	0.52
No	0.89 (0.57–1.39)	0.60	1.70 (1.22–2.37)	0.002	
**Chronic liver disease**					
Yes	0.81 (0.10–6.84)	0.85	1.81 (0.36–9.14)	0.48	0.27
No	0.90 (0.57–1.42)	0.65	1.54 (1.10–2.15)	0.01	
**Chronic immunosuppression**					
Yes	0.76 (0.31–1.87)	0.56	2.30 (1.02–5.22)	0.05	0.88
No	0.94 (0.56–1.57)	0.82	1.78 (1.24–2.55)	0.002	
**APACHE II score**					
≤ 23	0.58 (0.30–1.12)	0.10	2.22 (1.32–3.74)	0.003	0.05
> 23	1.43 (0.75–0.99)	0.28	1.48 (0.99–2.22)	0.06	
**Diabetes**					
Yes	1.06 (0.51–2.21)	0.87	1.59 (0.97–2.58)	0.06	0.38
No	0.80 (0.46–1.40)	0.43	1.55 (0.98–2.45)	0.06	
**Vasopressors**					
Yes	1.11 (0.61–2.05)	0.73	1.83 (1.22–2.73)	0.004	0.60
No	0.71 (0.37–1.37)	0.31	1.27 (0.73–2.23)	0.40	
**ICU duration (days)**					
≤ 5	0.86 (0.43–1.73)	0.68	2.29 (1.35–3.87)	0.002	0.06
> 5	0.93 (0.52–1.66)	0.81	1.31 (0.87–1.97)	0.20	
**Estimated GFR**					
≤ 73	0.94 (0.44–2.03)	0.88	1.92 (1.32–2.81)	0.0007	0.14
> 73	0.81 (0.47–1.40)	0.44	1.17 (0.58–2.35)	0.67	
**Acute Kidney Injury**					
Yes	0.73 (0.19–2.78)	0.65	1.74 (0.95–3.17)	0.07	0.63
No	0.91 (0.57–1.46)	0.71	1.52 (1.03–2.24)	0.03	
**Type of admission**					
Non-operative	0.85 (0.55–1.33)	0.48	1.66 (1.20–2.30)	0.002	0.70
Post-operative	6.50 (0.28–151.13)	0.24	1.63 (0.12–22.98)	0.72	

*aOR* adjusted odds ratio, *APACHE* Acute Physiology and Chronic Health Evaluation, *CI* confidence interval, *GFR* glomerular filtration rate, *ICU* intensive care unit

Furthermore, there were no significant differences in-hospital mortality in the selected subgroups patients as shown in Table 5.

## Discussion

Our study showed that hyperphosphatemia, but not hypophosphatemia, during the first 24 h of ICU admission was associated with an increase in-hospital mortality in critically ill patients with sepsis or septic shock.

Phosphate has various physiological functions. It is a vital component for intracellular metabolism and affects respiratory muscle contractility, neuronal transmission, and electrolyte transport. Furthermore, it has a role in supplying oxygen to tissues, maintaining plasma and urinary pH, coagulation cascade as well as body immune system [1]. Hence, low serum phosphate may interfere with all these physiologic processes and affect the outcomes of patients with hypophosphatemia. On the other hand, hyperphosphatemia can lead to the formation of calcium phosphate crystals, which in turn can cause vascular disease and organ dysfunction such as acute kidney injury [28].

Phosphate disturbances in critically ill patients is managed as per an approved protocol from the pharmacy and therapeutic committee at King Abdulaziz Medical City Riyadh. Also, it is available via order set in BESTCare. The protocol has addressed the management based on the phosphorus serum level concentration. In critically ill patients,  an acute increase in serum phosphorus levels will result in the formation of calcium phosphate crystals, which in turn can cause an acute kidney injury which further comprimises it clearance.

Studies that evaluated the association between hypophosphatemia and mortality had controversial results [1]. This could be due to several limitations such as low sample size, differences in settings and patient populations, and uneven cut-off points of phosphate concentration levels between the studies. Shore et al. conducted a retrospective study that compared severe hypophosphatemia (phosphate level < 1 mg/dl) versus hypophosphatemia (phosphate level > 1 mg/dl) and mortality in 55 patients with sepsis. They found that those with severe hypophosphatemia had significantly higher mortality rates (80.8% versus 34.5%; *p* = 0.001) [29]. Sankaran et al. conducted a retrospective study that reviewed the laboratory abnormalities of 302 patients who were admitted to ICU with bacterial pneumonia. They showed that hypophosphatemic patients experienced a higher mortality compared to normophosphatemic subjects (*p* < 0.001) [30]. Zazzo JF et al. evaluated 208 patients who were admitted to the surgical ICU over 6 months and found the mortality was higher in the hypophosphatemic group than in the normophosphatemic group (30% versus 15.2%; *p* < 0.05) [31]. In contrast, many other studies did not find any association between hypophosphatemia and mortality. Demirjian et al. reported a single-center prospective observational study in which 321 patients with acute kidney injury on continuous renal replacement therapy were included for the association of mortality. Hypophosphatemia occurred more frequently during dialysis but was not significantly associated with 28-day mortality (OR 1.16; 95% CI: 0.76–1.77) [18]. Lim C et al. analyzed data from a prospective cohort study of medical and surgical ICU patients with renal replacement therapy for acute kidney injury and found no significant difference in both ICU mortality and hospital mortality in patients with hypophosphatemia compared to patients without hypophosphatemia [28] Yang Y et al. retrospectively investigated hypophosphatemia in critically ill patients with acute kidney injury who received continuous venovenous hemofiltration, and also showed that hypophosphatemia was not associated with 28-day ICU mortality (*p* = 0.7) [29]. Suzuki et al. conducted a retrospective observational study that included generally critically ill patients and showed that hypophosphatemia was not associated with ICU mortality (aOR 0.86, 95% CI: 0.66–1.10; *p* = 0.24) and hospital mortality (OR 0.89, 95% CI: 0.73–1.07; *p* = 0.21) on multivariable logistic regression analysis. They concluded that hypophosphatemia was likely a marker of illness severity [13]. Haider et al. had supported this finding as well as they showed no association between hypophosphatemia and mortality in general critically ill patients who presented to the emergency room [14].

Few studies looked at the association between hyperphosphatemia and mortality. Indeed, their findings were consistent with our findings. Haider et al. studied the association between hospital mortality and phosphate level in unselected patients presenting at an emergency room. Their results showed that hyperphosphatemia was associated with a significant increase in hospital mortality (OR 3.29, 95% CI: 1.8–6.1, *p* < 0.001) [14]. In a post hoc analysis, Kuo et al. evaluated data of patients who were admitted to a burn unit and found that the 90-day mortality was higher in the hyperphosphatemia group (53.8% versus 18.1%, *p* < 0.001), and the difference was still significant even when adjusting for several confounding factors (hazard ratio 2.05, 95% CI: 1.17–3.59) [25]. Miller et al. evaluated 197 ICU patients who were hospitalized for severe sepsis or septic shock and on mechanical ventilation and found that 33 (16.7%) of them were hypophosphatemic, and 41 (20.8%) hyperphosphatemic. The mortality rate was significantly higher among those with hyperphosphatemia (*p* = 0.012) [22].

Hypophosphatemia is usually treated in the ICU using intravenous or oral phosphate administration. In our ICU, we use a protocol for the replacement of serum electrolytes including phosphate. Lower caloric intake in patients at risk for refeeding syndrome will probably reduce its occurrence and result in improved outcomes [32]. Adequate nutritional support in other patients may prevent and treat hypophosphatemia. For hyperphosphatemia, the management in critically ill patients is usually directed at its cause. Additionally, specific treatments include adequate hydration with diuresis in patients with normal kidney function, the reduction of phosphate intake by utilizing low phosphate feeding formulas, the use of phosphate binders and renal replacement therapy [33]. Whether these specific treatments improve the outcomes of critically ill patients is unknown.

Our study has both strengths and limitations. The strengths are, first, the inclusion of a larger sample size from different medical, surgical, trauma and neurocritical ICUs; and second, comparing 3 groups of phosphate concentration levels on the first day of ICU admission to avoid possible confounder factors such as therapeutic interventions of nutrition, phosphate binders, insulin, and catecholamines. On the other hand, the limitations of our study include its retrospective design and that data were collected in a single tertiary medical center which may limit the generalization of our findings. Hyperphosphatemia might be a marker of higher severity of illness and thus the association with higher mortality. However, we adjusted for APACHE II score in the multivariable logistic regression analysis model.

## Conclusion

In patients with severe sepsis and septic shock, hyperphosphatemia was associated with increased hospital mortality. Further studies are needed to clarify the impact of hypophosphatemia as well as hyperphosphatemia in critically ill populations. Early diagnosis and management are required to prevent their detrimental effects and improve the overall outcomes.

## Data Availability

The datasets generated and analysed during the current study are not publicly available but will be made available from the corresponding author on reasonable request.

## Abbreviations

ICU: Intensive care unit
APACHE: Acute Physiology and Chronic Health Evaluation
GCS: Glasgow Coma Scale
eGFR: Estimated glomerular filtration rate
INR: International Normalized Ratio
aOR: Adjusted odds ratio
CI: Confidence interval
